# Development of an equation to screen for solar hemorrhages from digital cushion ultrasound texture analysis in veal calves at slaughter

**DOI:** 10.3389/fvets.2022.899253

**Published:** 2022-07-29

**Authors:** Giorgia Fabbri, Luisa Magrin, Flaviana Gottardo, Leonardo Armato, Barbara Contiero, Matteo Gianesella, Enrico Fiore

**Affiliations:** Department of Animal Medicine, Production and Health (MAPS), University of Padua, Viale dell'Università, Legnaro, PD, Italy

**Keywords:** claw lesions, sole hemorrhage, texture analysis, ultrasound, veal calves

## Abstract

Claw disorders are a relevant welfare issue in the cattle industry, fast and accurate diagnoses are essential for successful treatment and prevention. The present study aimed to develop an equation to assess the presence of solar hemorrhages from real-time ultrasound images texture analysis at slaughter. Eighty-eight hind feet were collected at the slaughterhouse from 44 Holstein male veal calves. The claws were trimmed by a veterinarian hoof-trimmer, approximately 30 min after the calves' slaughter, and classified into healthy and affected by solar hemorrhages. At the same time, ultrasound images were collected for each claw. Sole soft tissues' thickness was measured, and texture analysis was performed using MaZda software. The resulting parameters from sole soft tissues' measurements and texture analysis were screened with a stepwise linear discriminant analysis using the absence or presence (0/1) of solar hemorrhages as the dependent variable. Results from the stepwise analysis identified 9 variables (among 279) as predictors, and an equation was developed and used to predict the presence or absence of solar hemorrhages on the scanned claws by binary measure: values ≤0.5 counted as 0, while those >0.5 as 1. Validation of the equation was performed by testing predicted lesions (LESpred) against the clinically evaluated lesions (LESeval) with a confusion matrix, a ROC analysis, and a precision-recall curve. Results of the present study suggest that the equation proposed has a good potential for detecting effectively hemorrhages of the sole by ultrasound imaging texture means, and could be used to monitor unsatisfactory housing and management conditions at the farm level, and for early management intervention and prevention.

## Introduction

The beef cattle production system in Europe is characterized by indoor confinement on concrete flooring systems and high-concentrate diets during the fattening phase. It has been proved that these intensive housing and feeding conditions implemented to maximize animal growth can predispose the animals to develop metabolic, joint, and claw disorders (1, 2). Veal calf production, another consistent subsystem of the European meat industry, is mainly based on the indoor fattening of young male calves of dairy breeds for about 6 months to reach an average carcass weight of at least 160 kg, which is much lighter than that of intensively finished beef cattle. A further specific trait of the veal calf industry is the paler color of the meat, due to the feeding plan mainly based on milk replacer and corn grain and low amounts of fibrous feed. Only recently, results obtained by a *post-mortem* organs inspection at the slaughter of several batches of veal calves (3) revealed that 65–97% of inspected claws per batch were affected by solar hemorrhages (SH). Such results were unexpected because of the young age of the investigated animals who were considered too young to be affected by foot diseases and are overlooked by research.

Solar hemorrhages (SH) are among the most frequently recorded claw-horn disruption lesions (CHDL) both in dairy and beef cattle (2, 4), a group of disorders that includes also white line hemorrhages, sole ulcers, and white-line fissures (5, 6). The development of CHDL, including SH, in dairy cattle is reported to be associated with ruminal acidosis conditions, resulting from the consumption of high-concentrate diets (7, 8), and they are known to significantly affect cow welfare and farm profitability (9–12). Veal calf's production relies on feeding with an even higher concentrates ratio, placing these animals at risk for claw disease development, despite their young age. Although SH is not frequently associated with clinical signs of lameness, extended and severe SH could coexist alongside other hoof lesions or lead to more debilitating ones over time since they are recognized being the first symptom of subclinical laminitis (13, 14). Cattle have higher productivity when free from diseases and pain, therefore, the development, evaluation, and application of protocols that prevent diseases such as claw disorders are essential to ensure the rearing of healthy and productive cattle (15). In particular, beef cattle affected by claw diseases tend to have slower growth curves than non-affected animals (1.75 vs. 2.95 lbs/day) (16), which results in prolonged fattening periods before the required slaughter weight reached, leading to higher feeding costs and more labor.

For successful treatment and prevention of CHDL, a fast and accurate diagnosis is necessary. It can reduce drug misuse and dosage, enhance recovery, and reduce losses. In the cattle industry, ultrasound (US) imaging has been used to investigate muscle and musculoskeletal disorders, and to measure the hoof capsule, corium, and soft tissues' thickness in bovine claws (17–19). It is considered a useful diagnostic tool to investigate rapidly and in real-time the deep structures of the bovine foot, and permitted on-field investigation of dairy cow digital cushion (20). Early detection of claw disorders by US investigation of soft tissue echogenicity could represent an important contribution to prevention and treatment. Several studies have linked the thickness of the sole's soft tissue to the development of CHDL in dairy cows (21). The soft tissue was thinner in claws from the cadavers of culled lame cows compared with non-lame cows (22). The thickness of the digital cushion measured using ultrasonography on elevated claws has also been associated with lameness and CHDL in a cross-sectional study (23). However, very little is known so far about the prevalence of solar hemorrhages in veal calves and their investigation with US imaging texture analysis. The present study aimed to elaborate a formula capable of screening the presence or absence of solar hemorrhages from real-time US images and texture analysis in veal calves through a *post-mortem* inspection at the slaughterhouse.

## Materials and methods

### Experimental design

The present study is part of a wider *post-mortem* inspection of the rumen, abomasa, liver, and claws of veal calves at slaughter (3, 24). It is focused on 44 apparently healthy Holstein male veal calves, inspected at slaughter in a commercial cattle slaughterhouse in the North of Italy. According to data gathered *a posteriori* from slaughter records, calves were slaughtered after 185 ± 16.7 (mean ± SD) fattening days and at an average carcass weight of 165 ± 26.6 kg. Based on the information provided by farmers, all calves inspected at slaughter were housed indoors in closed buildings equipped with mechanical ventilation systems and kept in multiple pens (4–5 calves) with fully-slatted wooden floors. The feeding program consisted of reconstituted milk replacer and solid feed, delivered two times a day. During the entire fattening, the amount of milk replacer and solid feed provided to calves was on average 312 and 162 kg, respectively, and the solid feed was composed of 85–93% corn grain (24).

### Post-mortem claws assessment and measurements

**Figure 1:**
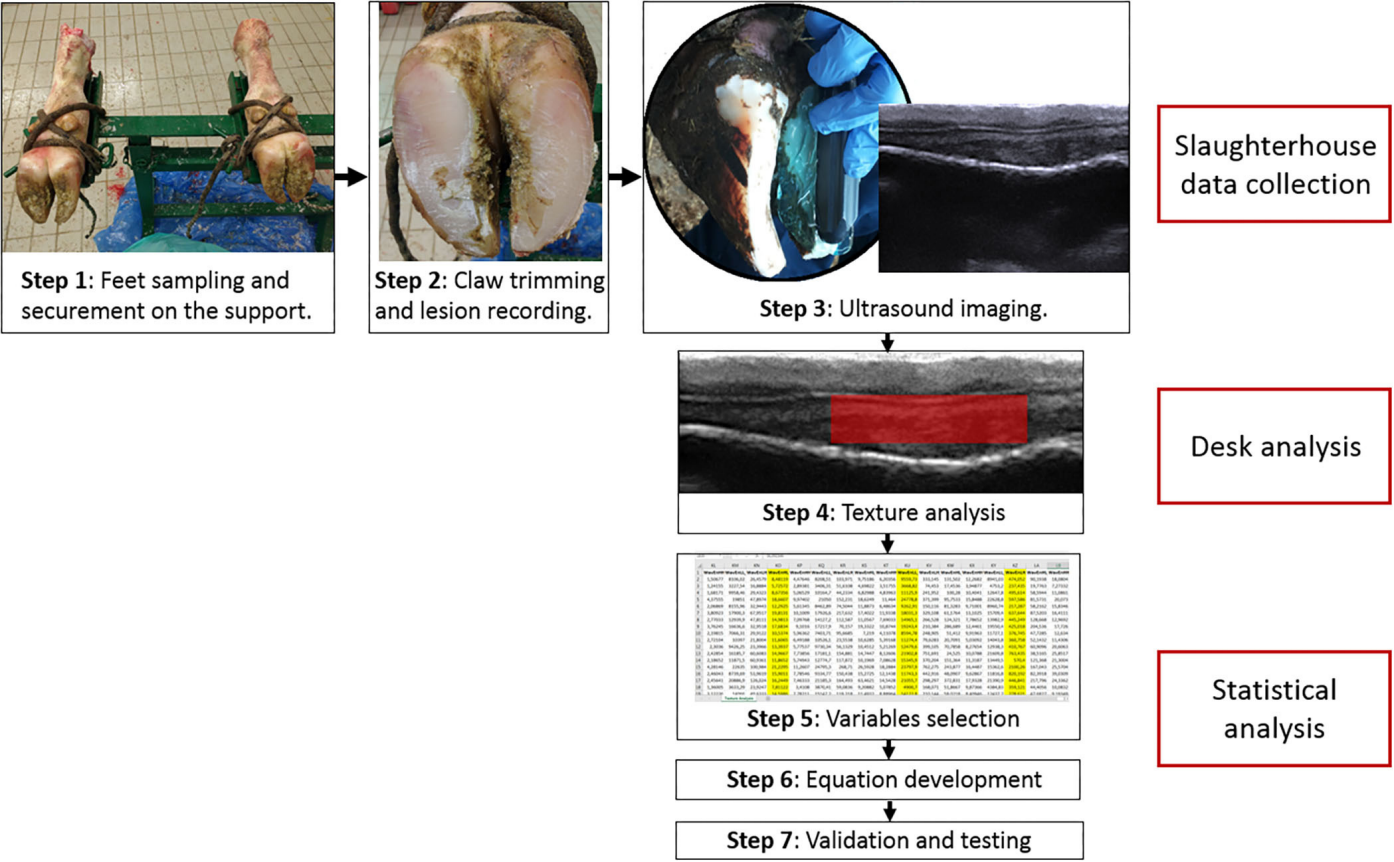
Operational flow chart of the methods in the study. Steps 1–3 were performed at the slaughterhouse and consisted in: securing the limbs to the support (step 1), claw trimming and lesion recording (step 2), ultrasound imaging (step 3). Step 4 was texture analysis with Mazda program on US images and measurement of S1, S2, and S3 sole soft tissue thickness. Steps 5–7 were performed statistically and consisted in: selection of the variables to include in the equation using stepwise analysis (step 5), development of the equation (step 6), and validation of the equation and performance testing using ROC, precision-recall curve and confusion matrix.

Detailed methodological procedures are described in Magrin et al. (3, 24). The distal anatomical part, from the hock to the claw, of both hind limbs of each calf was directly collected from the slaughter line by slaughterhouse operators after the stunning procedure and hanging of the carcass aloft. After taking the limbs out of the slaughter chain, samples were identified for further reference and secured on handmade support, as depicted in the methodological flowchart in Figure 1. Such handmade support was in the proximity of the slaughter chain and the time between animals' death and claws' evaluation was approximately 30 min. Claws' position on the handmade support reproduced precisely that claws would have had if they were trimmed on a living animal, contained in a regular trimming chute. After the proper placement, claws were then trimmed by a veterinarian with an electric grinder following guidelines from Toussaint Raven (25) for functional trimming. After trimming, claws were evaluated by the same veterinarian, and any sign of claw disorder or defect was registered, following the description of the International Committee for Animal Recording (ICAR) Claw Health Atlas (26). Claw disorders were recorded as a binary measure: presence or absence of solar hemorrhage. The inspection by the veterinarian was regarded to be the golden standard to validate the equation and will be termed as “LESeval” from here on. A total of 88 hind feet and 176 respective claws belonging to 44 Holstein male veal calves were inspected.

### Ultrasonographic images acquisition and analysis

Functional trimming removed loose horn charged with air and created a flat weight-bearing surface (27). Ultrasound examination was then performed using a portable ultrasound scanner (MyLabOne™, Esaote S.p.a., Genova, Italy) equipped with a multifrequency linear probe (SC3421, Esaote S.p.a., Genova, Italy; 7.5–10.0 MHz). The ultrasound settings were maintained constant throughout all scans: frequency 10.0 MHz, 7 cm depth acoustics window, 100% greyscale gain, and time-gain compensation in a neutral position. The probe was positioned on the plantar aspect of the foot, as described by Laven et al. (28) and Fabbri et al. (20). Ultrasound gel was used as a conductor between the sole horn and the transducer to ensure clear viewing of the sole soft tissues beneath the horn. A minimum of two US scans were collected for each investigated claw. The images were then stored in a BMP/JPEG format without compression for further texture analysis. The analysis on US images was performed blindly, and information on which claws were diagnosed as affected and which as healthy was not disclosed until the measurements on US images and the texture analysis had been performed. For each lateral and medial claw, a single ultrasound image was selected for sole soft tissues' thickness and texture analysis. Images underwent no preprocessing. The sole soft tissue thickness measurement was performed using MyLab™Desk software (SC3421, Esaote S.p.a., Genova, Italy), while texture analysis was performed using free purpose-specific software (MaZda v4.6; Technical University of Lodz, Institute of Electronics, Poland). Sole soft tissues' thickness was evaluated in three different sites (S1, S2, and S3) of the weight-bearing surface of each claw (27). The first measure (S1) of sole soft tissue thickness was taken perpendicular to the apical margin of the distal phalanx. The third measure (S3) was taken in the caudal margin of the third phalanx, perpendicular to the tuberculum flexorium. The second measure (S2) was between S1 and S3 sites, equidistant from the two sites (27).

One rectangular region of interest (ROI) was set manually on the area between the sole's internal surface and the plantar margin of the distal phalanx, avoiding any ultrasonographic artifacts to compute texture analysis. Texture analysis is a post-processing method for ultrasound images that provides parameters of texture features expressed as numerical values that quantitatively evaluate images. These texture features include histogram, gradient, gray-level co-occurrence matrix, run-length matrix, autoregressive model, and wavelet transform parameters. The average ROI size was 14,149.13 pixels ± 3,099.63 SD, and Mazda software analysis produced automatically 279 descriptors that characterize a gray-scale image texture. These descriptors can be grouped into 6 main categories: gray-level histogram features (*n* = 9, the basic characters reflecting image uniformity), gradient features (*n* = 5, a direction change in gray-level intensity representing the image intensity distribution), the run-length matrix (*n* = 20, calculated from different run angles in four directions including horizontal, vertical, 45°and 135°, and indicating image coarseness), the co-occurrence matrix (*n* = 220, computed from intensities of pairs of pixels and describing the homogeneity), the autoregressive model (*n* = 5, the coefficients of neighboring pixels reflecting the coarse-to-fine stratification), and the wavelet transform (*n* = 20, the spatial frequencies at multiple scales identifying coarseness). Texture analysis was performed following the software developer indications (co-occurrence matrix: six bits/pixel, gradient features: 8 bits/pixel, run-length matrix: four bits/pixel, and wavelet transform: 12 bits/pixel) (18, 19).

### Statistical methods

All statistical analysis was carried out using commercially available statistical software SAS 9.4 (SAS Inst. Inc., Cary, NC, USA). Texture parameters and sole soft tissues' thickness were screened with a stepwise linear discriminant analysis using the absence or presence (0/1) of solar hemorrhages as the dependent variable and the outcomes of ultrasound imaging analysis as explanatory variables. The goal was the identification of the best high-quality combinations of variables to create a parsimonious model with as few parameters as possible to enhance stability during validation. The regression equation capable of maintaining the highest number of variables and, at the same time, keeping the variance inflation factor (VIF) lower than 10 for all included variables was assumed as a predictive model. The equation resulting from such analysis was used to predict normal or abnormal images (presence or absence of solar hemorrhages, respectively) of the scanned claws. The outcome of the formula was converted into a binary measure: all values ≤0.5 were counted as 0, while those >0.5 were counted as 1. Predicted Lesion (LESpred) was then tested against clinically evaluated lesion (LESeval) by means of a confusion matrix to compute sensitivity, specificity, accuracy, precision, and misclassification rate. A ROC curve and a precision-recall curve were also performed.

## Results

Sole hemorrhage was the only disorder detected on affected claws, except for a single case of white line lesion that was therefore excluded from the statistical analysis. Among the 175 evaluated claws, 61 (35%) were healthy, and 114 (65%) were affected by solar hemorrhage. Fifty-nine sole hemorrhages (52%) were found on the lateral claw, and 55(48%) on the medial one. The distribution of the affected claws was similar between the right and left feet: 59 (52%) on the right foot and 55 (48%) on the left foot. Among all the considered parameters, results from the stepwise analysis indicated 9 variables as predictors. The selected parameters were: the S3 measure, regarding the thickness of sole soft tissues near the heel; MaxLum and MinLum, maximum and minimum brightness values of the images; S(3,3)InvDfMom, S(0,4)InvDfMom, and S(4,0)DifVarnc which all belong to the co-occurrence matrix category; and WavEnHL_s-2, WavEnLL_s-4, and WavEnLH_s-5 which belong to the wavelet transform category. The obtained variables were assembled into a model that had a VIF below 10 for all the included parameters and was carried out for development and testing.

The resulting regression equation was as follows:


$$\begin{array}{l} LESpred\;=\;-1.29377+(0.00981\;*\;MinLum)\;\\ \;+(0.00974\;*\;MaxLum)\;+\;(0.10477\;*\;S3)\;\\ \;+\;(13.61477\;*\;S(3,3)InvDfMom)\;\\ \;+\;(-0.29096\;*\;S(4,0)DifVarnc)\;\\ \;+\;(-16.94933\;*\;S(0,4)InvDfMom)\;\\ \;+\;(0.07444\;*\;WavEnHL\_s-2)\;\\ \;+\;(-0.00002446\;*\;WavEnLL\_s-4)\;\\ \;+\;(0.00018946\;*\;WavEnLH\_s-5) \end{array}$$

**Figure 2:**
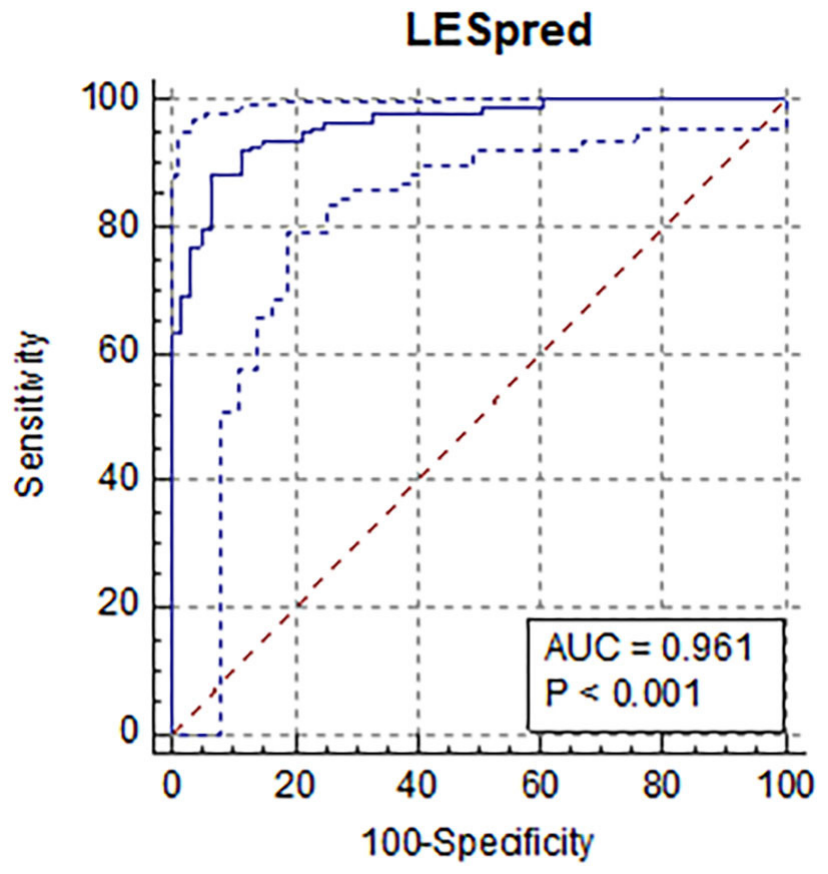
Results of the receiver operator curve (ROC) for the results of the prediction equation showing an area under the curve of 96% (AUC=0.961; 95% CI: 0.55–0.69; positive likelihood ratio = 13.5) using an optimal cut-off value of 0.61, sensitivity was 88.6%, and specificity was 93.4%. All the variables included were statistically significant.

**Figure 3:**
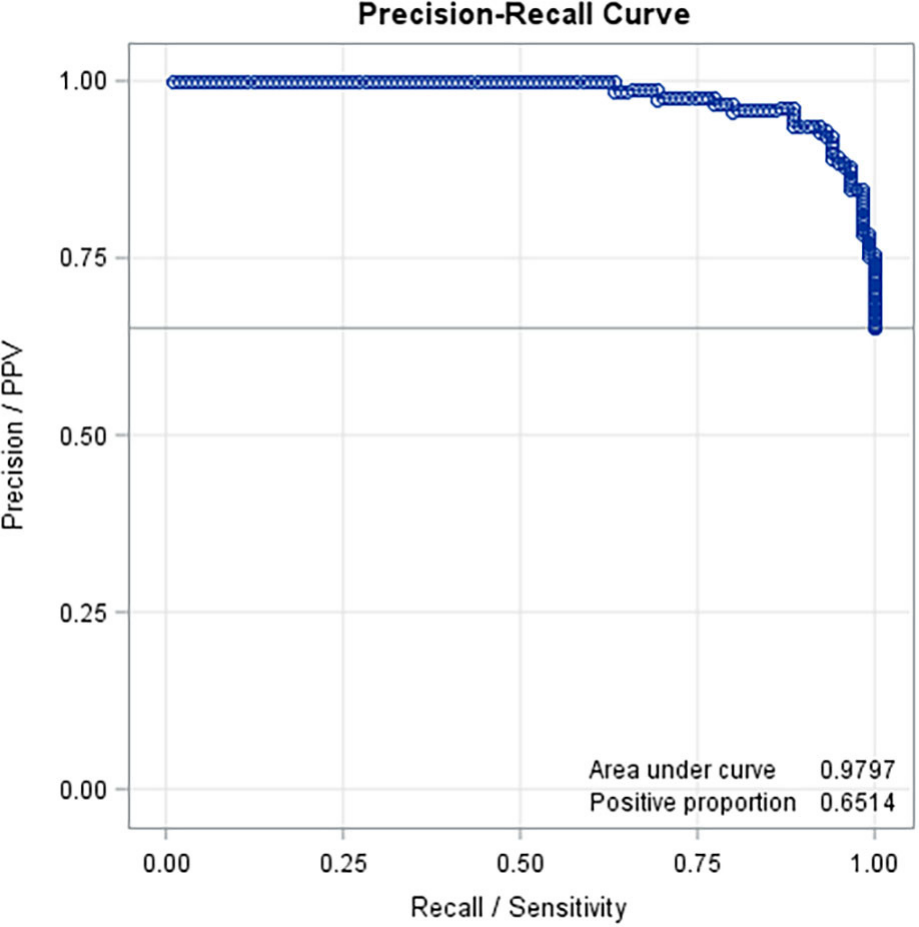
Precision-recall curve based on the results of the prediction equation obtained with the variables selected with the stepwise method (AUC = 0.97).

Predicted values (both for normal and abnormal US images) ranged from −0.26 to 1.53. The mean predicted value for healthy claws was 0.27 (SD = 0.24; min = −0.26, max = 0.78); whereas for the affected claws was 0.85 (SD = 0.24; min = 0.23, max = 1.53). Results from the confusion matrix and misclassification rate are reported in Table 1, a comparison between the sole hemorrhages observed by trimming and those predicted by the equation is summarized in Table 2 and examples of US image classification are provided in Supplementary file 1. Moreover, the ROC analysis is reported in Figure 2 and precision-recall curve in Figure 3.

**Table 1:** Results from the confusion matrix on LESpred (estimated using the 0.5 cutoff on the results from the prediction equation) tested against LESeval (clinically evaluated lesion) indicating the sensitivity (proportion of true positive cases that were correctly identified), the specificity (proportion of true negative cases that were correctly identified), the accuracy (proportion of correctly identified cases as both true positive and true negative), the precision (proportion of correctly identified true positive cases belonging to the actual (true positive and false positive) group), and the misclassification rate of the proposed method.

**Indices**	**Predicted values**
Sensitivity (95% CI)	0.93 (0.88–0.96)
Specificity (95% CI)	0.85 (0.79–0.90)
Accuracy	0.90
Precision	0.87
Misclassification rate	9.71

95% CI, 95% confidence interval.

**Table 2:** Comparison between the visually assessed sole hemorrhages (LESeval), observed during trimming, and those predicted by the equation (LESpred).

	**LESeval**	**LESpred**	
		**Correctly identified**	**Wrongly identified**
Healthy	61	52	9
Affected	114	106	8
Total	175	158	17

## Discussion

The prevalence of sole hemorrhages detected on the claws of veal calves after a random *post-mortem* inspection at slaughter was rather high, reaching 65% of all evaluated claws. Considering the low slaughter weight of these animals, this could probably be due to the high corn grain diet, which could predispose them to a subclinical acidosis condition and negatively impact claws' health. Circulatory disturbances, represented by acute focal hemorrhages, have a higher incidence than all other claw disorders, and were the main detected lesions in male calves at 26 weeks of age (29) and in studies on dairy cattle (4). However, sole hemorrhages seem to have little to no effect on locomotion, suggesting that these specific disorders do not appear noticeable with a lame gait (30). This could be the reason why the distribution of sole hemorrhages observed *post-mortem* ranges so high in the population of veal calves without being detected or even suspected *in vivo*.

The distribution of solar hemorrhages was similar between lateral and medial claws, unlike what is usually stated in dairy cows' literature, where lateral digits are described as more affected than medial ones (31, 32). Such disparity is attributed to the asymmetry between the left and medial claw (especially in the hind limbs), and because lateral claws bear higher weight than the medial ones, which could predispose the lateral claw to disease development (31, 32). However, a study from Parés-Casanova et al. (33) showed no difference in size between medial and lateral distal claw surfaces on 9–15 months old calves. The calves inspected in the present study were younger than those investigated by Parés-Casanova et al. (33) and had not reached their full growth both in terms of weight and locomotory apparatus conformation. It is possible that such difference between lateral and medial digits had not been expressed yet enough to create a difference in weight bearing and lesion insurgency.

A well-developed digital cushion is necessary to reduce and prevent contusions that lead to claw lesions (15) and the presence of solar corium hemorrhage and swelling can represent the outset of the problem and lead to secondary white line separation and infection (34). Overgrown and malformed claws are vulnerable to mechanical injury and penetration by infectious agents; thus, this condition may easily lead to bigger disturbances in animals' overall health and locomotor apparatus (35). For example, a study conducted by Drendel et al. (36) found that heifers affected by claw disorders at a young age had a high incidence of having reoccurring claw disorders later in life. Such a high prevalence of SH in young veal calves can be considered an alarming factor for beef cattle rearing, because it could be a predisposing factor for future lameness problems in heavier animals (37). Compared with non-lame cattle, lame cattle are frequently culled prematurely and have a lower carcass weight, conformation class, and fat coverage, which impair carcass value (38–40). For such reasons, prevention and early identification and treatment of lame cattle can reduce culling rates and improve carcass value.

Studies investigating the correlation between solar soft tissue thickness and CHDL found that thinner solar soft tissues were correlated with CHDL insurgency (41, 42). In the present study only S3 was correlated with solar hemorrhage but, as stated before the animals in the present study were very young and had not reached yet full growth of their pedal structures. Unfortunately, the abovementioned studies did not consider texture analysis in addition to the solar soft tissue thickness to permit comparison. One of the problems when working with feature extraction methods for texture analysis is the multitude of generated features. As the number of features increases, the classification model becomes more complex and the classification performance decreases, because of the redundant or irrelevant features that inhibit the classification performance (43). Choosing the most relevant features to detect the target lesion is mandatory to create reliable models. Reducing the number of features not only increases classification performance but also speeds up the testing of new data and makes the classification problem easier to understand (44, 45). In the present study, 9 features were selected to be included in the model the S3 measure, regarding the thickness of sole soft tissues near the heel; MaxLum and MinLum, maximum and minimum brightness values of the images; S(3,3)InvDfMom, S(0,4)InvDfMom, and S(4,0)DifVarnc which all belong to the Co-occurrence matrix category; and WavEnHL_s-2, WavEnLL_s-4, and WavEnLH_s-5 which belong to the wavelet transform category. The parameters in the present study can therefore be reverted to 4 main categories: one parameter was related to anatomical measures; two parameters were related to image brightness; three parameters were related to co-occurrence matrix; and 3 parameters were related to wavelet transform category. The measure of sole soft tissue thickness in S3 seems to be highly predictive of foot lesions in veal calves, while the same measure in S1 and S2 did not have the same discernment power. This is partially concordant with studies from Toholj et al. and Newsome et al. (40, 41) which stated that the sole soft tissue thickness is affected by CHDL while is in contrast with a previous study from Fabbri et al. (20) that found no differences for neither of the three digital cushion thickness measures between healthy cows and cows affected by claw diseases. This last study (20) suggested that it could be the composition of the digital cushion, and therefore the percentage of connective and adipose tissue that plays an important role in claw horn lesions insurgency. However, it is possible that lactating dairy cows, which receive functional trimming two times per year, have a better distribution of their weight on the whole claw sole, compared to veal calves which have never received any trimming and are reared on the harder floor, and therefore could be dissipating the weight more on the bulbar part of the foot.

Co-occurrence matrix features give statistical information regarding the distribution of pixel pairs in the image. With co-occurrence matrix extraction, pairs of pixels separated by a predefined distance and direction are counted. The count is based on the number of pairs of pixels that have the same distribution of gray-level values, and the resulting values are allocated in the co-occurrence matrix (46). Texture features based on co-occurrence matrices have become a popular method and have been proven to be useful for the classification of tissues and lesions in MRI (47, 48).

Wavelet energy (WavEn) feature is computed at 5 scales within 4 frequency bands: low-pass filtering in both directions (LL) assessed the lowest frequencies, low-pass filtering followed by high-pass filtering (LH) assessed horizontal edges, high-pass filtering followed by low-pass filtering (HL) assessed vertical edges, and high-pass filtering in both directions (HH) assessed diagonal details (49). The wavelet transform technique analyzes the frequency content of an image within different scales and frequency directions, and the frequency is directly proportional to the gray-level variations within the image. This is a useful technique in image processing to accurately analyze the abrupt changes in the image that will localize means in time and frequency (43). Wavelet-derived texture features have high discriminatory power and usually provide a good classification method (43). It was also demonstrated that wavelet texture features are less sensitive to changes in the MRI acquisition protocol (50).

We hope that ultrasound texture analysis technology can be developed in the future to the point that the procedure proposed in the present study could be incorporated into portable ultrasound machines to compute rapidly and in the field whether animals are developing foot diseases, thus permitting early intervention. However, several challenges are related to ultrasound scanning and texture analysis. Animals in the present study were young veal calves, and since the aging process can affect sole soft structure dimension and composition the proposed technique could be effective only when applied to calves of similar age to those in the study. Moreover, one of the limits of the present study could be that a small deterioration of limbs could have occurred since US scans were performed *post-mortem*. Although the time interposed between slaughter and US scans was kept at a minimum (<30 min), it is possible that *post-mortem* degeneration processes could already have started to occur, thus changing slightly soft tissue composition (e.g., bleeding out could have diminished the water content of highly perfused tissues). For such reasons, further research is needed to assess both live animals and different animals' ages.

In the present study, the distribution of SH was relatively high in the population since more than half of the investigated claws were affected, but no notable differences between lateral and medial claws were present. An equation was developed using nine variables selected by stepwise analysis and permitted screening for SH presence using sole soft tissue texture analysis. Results from the confusion matrix, ROC analysis, and precision-recall curve suggest that the technique proposed in the present study has a good potential for detecting effectively hemorrhages of the sole by ultrasound imaging texture means, thus permitting early interventions and prevention.

## Data availability statement

The raw data supporting the conclusions of this article will be made available by the authors, without undue reservation.

## Ethics statement

Ethical review and approval was not required for the animal study because the study was conducted on animals post-mortem at the slaughterhouse. All animals were slaughtered in accordance to EU regulations [Council Regulation (EC) No 1099/2009 of 24 September 2009 on the protection of animals at the time of killing].

## Author contributions

GF: design of methodology, data curation, and original drafting. BC and LM: data curation and preprocessing. LA, LM, and FG: data collection. EF, FG, LM, and MG: design of methodology, draft reviewing, and editing. FG: funding acquisition. All authors contributed to the article and approved the submitted version.

## Funding

This research was funded by the University of Padova, Italy, grant number: PRAT project [CPDA158107].

## Conflict of interest

The authors declare that the research was conducted in the absence of any commercial or financial relationships that could be construed as a potential conflict of interest.

## Publisher's note

All claims expressed in this article are solely those of the authors and do not necessarily represent those of their affiliated organizations, or those of the publisher, the editors and the reviewers. Any product that may be evaluated in this article, or claim that may be made by its manufacturer, is not guaranteed or endorsed by the publisher.
