# Supplementary material for: Development of an equation to screen for solar hemorrhages from digital cushion ultrasound texture analysis in veal calves at slaughter
Source: Front Vet Sci. 2022 Jul 29;9:899253. doi: 10.3389/fvets.2022.899253 (PMC9372481; doi:10.3389/fvets.2022.899253)
Supplement: Supplementary file 1 [file Data_Sheet_1.docx]

Supplementary materials 1: Table

**Examples of US images classified by the prediction formula as “Normal” or “Abnormal” falling in the categories True positive, True negative, False positive, and False negative.**

The following images were analyzed using the prediction formula and were classified as normal or abnormal. Four examples for each True Positive (affected claws, classified as abnormal); True Negative (healthy claws, classified as normal); False Positive (healthy claws, classified as abnormal); and False Negative (affected claws, classified as normal) classification are provided, with the outcomes of the equation for each image.

As previously reported in materials and methods, the outcome of the formula was converted into a binary measure: all values ≤ 0.5 were counted as 0, while those > 0.5 as 1. Therefore, all images with values ≤ 0.5 were considered “Normal”, and all images with values > 0.5 were considered abnormal.

| **True Positive** | **True Negative** |
| --- | --- |
| 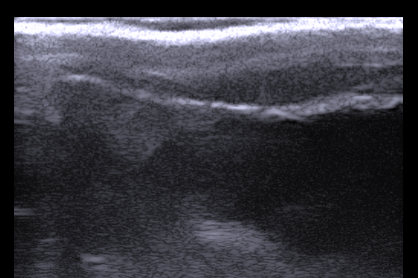 | 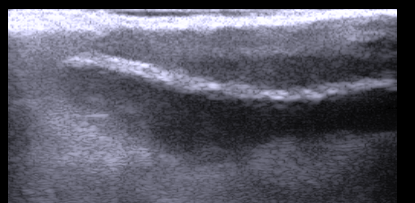 |
| **Image A:** Y = 0,87 | **Image B:** Y = 0,35 |
| 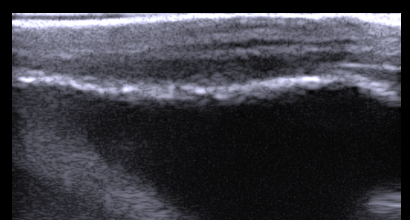 | 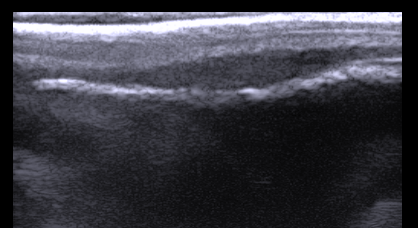 |
| **Image C:** Y = 0,93 | **Image D:** Y = 0,001 |
| 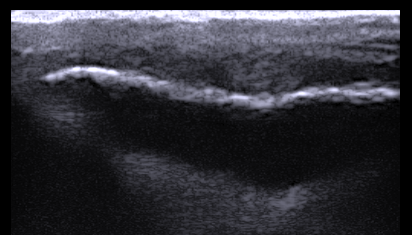 | 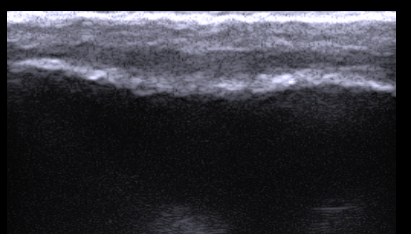 |
| **Image E:** Y = 0,77 | **Image F:** Y = -0,01 |
| 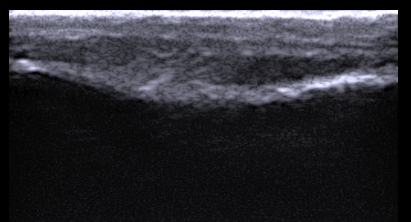 | 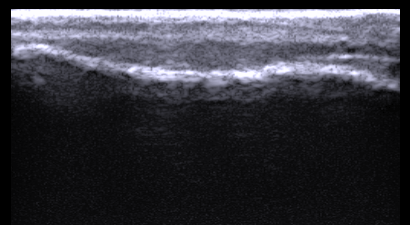 |
| **Image G:** Y = 0,80 | **Image H:** Y = 0,46 |

| **False Positive** | **False Negative** |
| --- | --- |
| 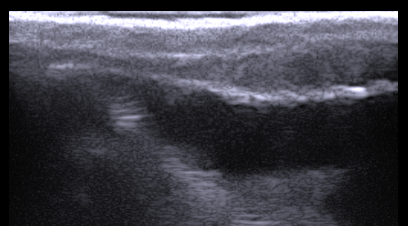 | 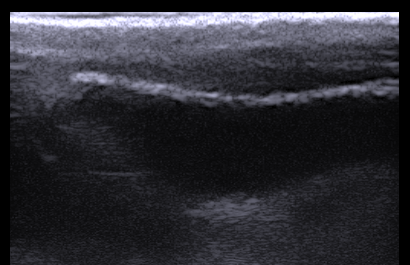 |
| **Image I:** Y = 0,74 | **Image J:** Y = 0,45 |
| 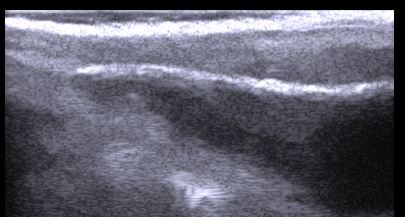 | 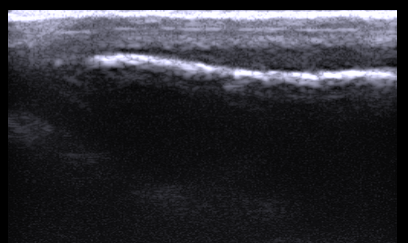 |
| **Image K:** Y = 0,59 | **Image L:** Y = 0,38 |
| 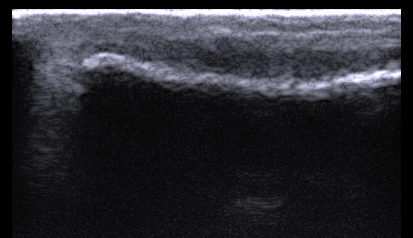 | 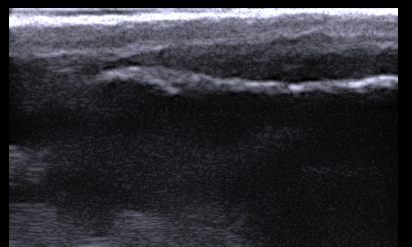 |
| **Image M:** Y = 0,51 | **Image N:** Y = 0,38 |
| 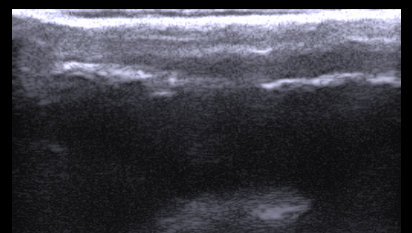 | 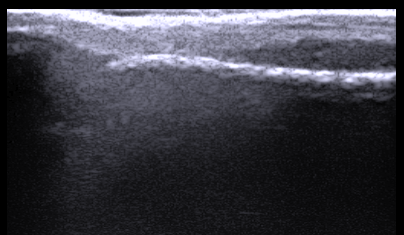 |
| **Image O:** Y = 0,78 | **Image P:** Y = 0,42 |
